# A systematic review of GWAS on CMR imaging traits: genetic insights into cardiovascular structure, function, and diseases

**DOI:** 10.1016/j.ebiom.2025.105992

**Published:** 2025-10-30

**Authors:** Sakina Lakda, Rhodri Huw Davies, Aroon Dinesh Hingorani, Nikhil Paliwal

**Affiliations:** a UCL Medical School, London, UK; b Institute of Cardiovascular Science, University College London, London, UK; c British Heart Foundation Research Accelerator, University College London, London, UK

**Keywords:** Genome-wide association studies (GWAS), Cardiac magnetic resonance imaging (CMR), Cardiac structure and function, Cardiovascular disease (CVD)

## Abstract

**Background:**

Cardiac magnetic resonance (CMR) imaging enables precise quantification of cardiac structure/function in biobanks, facilitating genetic investigation of cardiovascular traits. Genome-wide association studies (GWAS) have emerged as a useful tool to identify common and rare genetic variants associated with cardiovascular diseases (CVDs).

**Methods:**

We conducted a systematic review of CMR-based cardiac trait GWAS to generate an overview of phenotypes evaluated, loci identified and relationships with CVD outcomes.

**Findings:**

We identified 149 risk loci associated with chamber-specific cardiac traits, revealing fundamental insights into cardiac genetic architecture. Our findings demonstrate that ventricular structure and function are predominantly governed by genes such as *TTN* (identified in 47.1% of studies) and *BAG3* (29.4%) showing consistent associations across both left and right ventricular traits. In contrast, aortic measures were strongly associated with *ELN* variants (23.5%), highlighting specialised genetic control of vascular properties. Notably, several genes influence multiple cardiac chambers, with *TTN* variants associated with both left and right ventricular volumes and function, while *BAG3* and *PTPN11* regulate biventricular contractility, and *TBX5* impacts ventricular and atrial development.

**Interpretation:**

By identifying key genetic variants linked with CMR-derived cardiac traits, this review enhances our understanding of the genetics of CVDs and other medical conditions.

**Funding:**

British Heart Foundation Accelerator Award (AA/18/6/34223) and NIHR University College London Hospitals Biomedical Research Centre.

**Research in context**

**Evidence before this study**

Cardiac magnetic resonance (CMR) imaging offers gold-standard measurements of cardiac morphology and function and is increasingly used in large population biobanks. In recent years, genome-wide association studies (GWAS) using CMR-derived traits have identified common and rare genetic variants influencing cardiac structure and function. However, these studies have typically focused on specific cardiac chambers or traits in isolation. To date, there has been no comprehensive synthesis of the genetic loci identified across all cardiac chambers using CMR-derived phenotypes, nor a cross-comparison of the implicated genes or their relevance to cardiovascular disease outcomes.

**Added value of this study**

This is the first systematic review of GWAS conducted using CMR-derived cardiac phenotypes across the heart. By examining 17 studies that together report 149 significant loci, we provide a chamber-wise overview of genetic associations and identify key genes that are recurrently linked with cardiac traits. Our findings show that while genes such as *TTN* and *BAG3* are consistently associated with ventricular traits, vascular traits are more specifically linked to *ELN*. Furthermore, we demonstrate that several genes influence multiple cardiac chambers, supporting the existence of shared genetic pathways in cardiac development and function. We also highlight an overrepresentation of studies and associations involving ventricular traits, which may reflect both technical advantages of CMR in quantifying ventricular function and current research focus.

**Implications of all the available evidence**

Our synthesis underscores the potential of CMR-based GWAS to identify fundamental genetic determinants of cardiac structure and function. It highlights both chamber-specific and shared genetic influences and provides a framework for understanding how these loci relate to clinical cardiovascular diseases. These findings support the rationale for future integrative genetic studies and could inform the development of precision medicine approaches in cardiology. Additionally, the uneven distribution of chamber-specific data points to a need for more comprehensive studies evaluating atrial and vascular phenotypes to ensure balanced insights into cardiac pathophysiology.

## Introduction

Cardiovascular diseases (CVDs) are the leading cause of morbidity and mortality worldwide, accounting for approximately 17.9 million deaths annually, which equates to 31% of all global deaths.^1^ The substantial burden of CVDs on healthcare systems underscores the need for precise diagnostic tools and effective therapeutic strategies. In recent years, genome-wide association studies (GWAS) have revolutionised our understanding of the genetic underpinnings of complex diseases, including CVDs.^2^ The GWAS approach involves identifying genetic variants associated with CVDs by scanning the genomes of large populations to find genetic markers linked to disease traits. These studies have elucidated numerous genetic loci that contribute to the risk and progression of various cardiovascular conditions. For instance, significant associations have been found between genetic single nucleotide polymorphisms (SNPs) and diseases such as coronary artery disease (CAD), myocardial infarction (MI), and atrial fibrillation (AF).^3^, ^4^, ^5^

Cardiac magnetic resonance (CMR) imaging, a non-invasive technique that allows detailed characterisation of cardiac anatomy and function, can assess cardiac chamber volumes, systolic function, myocardial mass, and other structural parameters with high precision and reproducibility in population cohorts.^6^ The integration of CMR with GWAS has enabled a deeper understanding of the genetic determinants of cardiac morphology and function.^7^, ^8^, ^9^, ^10^ GWAS focussing on CMR-derived traits have identified several genetic variants associated with specific cardiac traits relating to the aorta and the 4 chambers. Genetic associations with cardiac chamber-specific traits have recently been studied, including titin (*TTN*) and BCL2-associated athanogene 3 (*BAG3*) variants influencing left ventricular volumes and systolic function, while atrial volumes are associated with Ankyrin Repeat Domain 1 *(ANKRD1)* and myosin light chain 4 (*MYL4*) variants that also predispose to AF and cardiomyopathies.^9^^,^^11^, ^12^, ^13^ Despite these advancements, these GWAS are limited by their focus on specific cardiac traits or a small subset of CVDs. Thus, there is a significant gap in understanding the comprehensive genetic architecture that links various cardiac traits within, and relationships to disease outcomes.

We therefore performed a systematic literature review of GWAS on CMR-based cardiac traits to create an atlas of genetic associations with cardiac shape, size, and function and investigated reported relationships with CVDs. By integrating and examining genetic association data for various cardiac traits, we sought to provide a holistic view of the genetic determinants influencing cardiac traits and investigate relationships of identified loci with CVDs. This comprehensive approach addresses the existing research gaps and contributes to the broader application of GWAS findings in clinical practice. Our hypothesis posits that specific genetic variants identified through GWAS on CMR traits are consistently associated with key cardiac functions and that some influence the risk of diseases. We focused on four key questions:

1. Which of the possible cardiac structure and function measures available from CMR have been the subject of a GWAS?
2. What are the most common genes associated with left ventricular (LV), right ventricular (RV), left atrial (LA), right atrial (RA), and aortic traits?
3. Are there common genes that appear across different cardiac chambers and traits?
4. How do the identified genetic variants relate to broader cardiovascular, cerebrovascular and other diseases?

## Methods

**Fig. 1:**
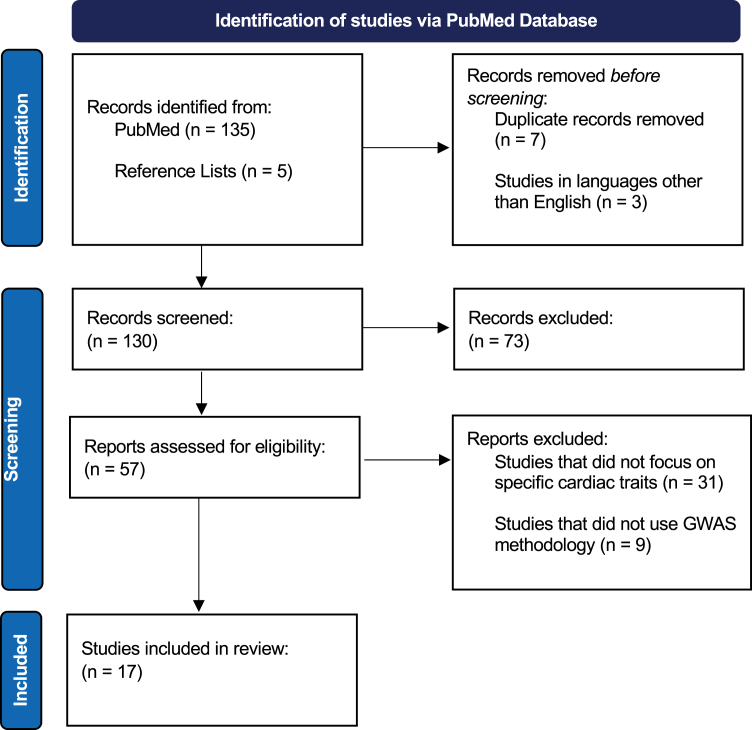
Study design based on the PRISMA flowchart.

The systematic review study design as shown in Fig. 1 encompasses the main steps: literature search, analysis and filtering, and review analysis. Detail on each step is provided in the following sections.

### Ethics

No ethical approval was required for the conduct of this systematic review because no primary data collection involving human or animal subjects took place, and the data and analyses presented are based on previously published peer-reviewed research.

### Search strategy

A comprehensive literature search was conducted using the PubMed and Embase databases to identify relevant studies involving GWAS on CMR traits. Our systematic review follows the PRISMA guidelines.^14^ The following Boolean search strategy was used:

*(“cardiac magnetic resonance” OR “cardiac MRI” OR “CMR”)*
*AND (“genome-wide association study” OR “GWAS”)*
*AND (“genetic”)*

The initial search yielded 135 records. Additionally, we manually reviewed the reference lists of relevant studies to identify 5 additional records, resulting in a total of 140 CMR-based cardiac trait records.

### Inclusion & exclusion criteria

The selection process involved two steps: analysis and filtering based on reading the full text of publications and the application of inclusion and exclusion criteria. The following criteria were used for including a record in our analysis:

• Date Range: The search included studies published up to April 2024 to ensure a thorough review of the available literature.
• Language: Only studies published in English were included to maintain consistency and ensure accurate interpretation of the findings.
• Types of Studies: The search targeted peer-reviewed articles that reported on cardiac MR-derived traits and GWAS-based genetic analyses.

The following criteria were used to exclude records:

• Studies not meeting the inclusion criteria were excluded.
• Duplicate records and studies without full-text availability and/or public GWAS summary statistics were excluded.
• Studies that did not focus on the specified cardiac traits or did not use GWAS methodology were excluded.

Out of the 140 identified records, 123 were excluded after reading the full text based on the inclusion and exclusion criteria. This rigorous filtering process ensured that only the most relevant studies were included in the review. A total of 17 records met the inclusion criteria and were included in the final review analysis.

### Study details & cardiac traits assessed

For each study, SL extracted key details including the author(s), publication year, sample size, and population demographic. The cardiac traits evaluated in each study were documented, including structural and functional measures. These traits were categorised by cardiac chamber (LV, RV, LA, RA, and aorta) to facilitate chamber-specific analysis.

### Identification of genetic variants associated with cardiac structure

The included studies were analysed to identify SNPs associated with chamber-specific cardiac traits. Each study's GWAS results were reviewed to document SNPs linked to distinct cardiac chamber traits.

### Assignment of SNP associations to genes

After identifying SNPs, we mapped them to genes using positional mapping, expression quantitative trait locus (eQTL) data, and gene-based association testing using Multi-marker Analysis of GenoMic Annotation (MAGMA) through Functional Mapping and Annotation (FUMA) software.^15^, ^16^, ^17^ To prioritise candidate genes, we applied a scoring system: one point for positional mapping, one point for eQTL support (with an extra point if the eQTL was in cardiovascular tissues such as heart, aorta, ventricle, or artery), one point for significant MAGMA gene–level association (P < 5 × 10^−8^), and an additional point if the nearest gene matched the FUMA-annotated gene.^18^ This approach ensured that genes supported by multiple lines of evidence were given higher priority.

### Prevalence of genes in cardiac chambers

An overall count was performed to identify which genes are most associated with each cardiac trait. A chamber-wise gene count was also performed across the different studies included, to identify the frequency of their associations reported with each cardiac chamber. Furthermore, we also identified genes that were common across more than one cardiac chamber (e.g., LV and RV).

### Association with cardiovascular diseases and other medical conditions

For the genes associated with cardiac chamber traits, we perform lookups for associations with CVDs in the GenCC and GWAS Catalogue databases.^19^^,^^20^ The association analysis aimed to highlight genes that not only influence cardiac traits but also contribute to the risk or progression of CVDs.

Additionally, we searched for links between the identified genetic variants with cerebrovascular diseases, as well as other relevant diseases of interest. These associations were explored by examining the overlap between genetic markers identified in the included GWAS and those reported in literature related to cerebrovascular and medical diseases. The GenCC database and GWAS Catalogue were further utilised to achieve this. Our analysis aimed to provide a broader understanding of the implications of the identified genetic variants and will be discussed further in the Discussion section.

### Statistics

No statistical analysis was performed as part of our methodology in this study.

### Role of funders

The funders had no role in the design of the study, data collection, data analysis, data interpretation, or writing of the study.

## Results

### Overview of studies and cardiac traits included

Our systematic review analysed 17 CMR GWAS encompassing individual study sample sizes ranging from 6765 to 43,230 individuals of predominantly European ancestry (Table 1). These studies collectively examined most of the major cardiac structures, with LV-based traits being the most frequently investigated (10 studies), followed by aortic (4 studies), RV (3 studies), and atrial measurements (2 studies for LA and 1 for RA). The largest GWAS was performed by Pirruccello et al. (2023) assessing aortic strain in 42,342 participants and Khurshid et al. (2023) evaluating LV mass index in 43,230 participants.^21^^,^^22^ The included studies measured comprehensive cardiac traits including ventricular volumetric parameters (end-diastolic volume, end-systolic volume, and ejection fraction), structural characteristics (myocardial mass, wall thickness, and trabeculations), and functional measures (aortic distensibility and strain, atrial emptying fractions). For example, Ahlberg et al. focused on left atrial volumes and emptying fractions in 35,658 participants,^12^ while Schmidt et al. examined biventricular volumes in 36,548 cases.^13^

**Table 1:** Summary of studies reporting genetic associations with CMR traits, organised by cardiac chamber(s) studied, sample size, key phenotypic measures, and risk of bias assessment.

First author (year)	Cardiac chamber(s) studied	Source	Sample size (n)	Key traits assessed	Risk of bias
Pirruccello et al. (2022)^21^	LV, RV, RA	UK Biobank	41,135	EDV, ESV, EF, SV	Low
Aung et al. (2022)^9^	RV	UK Biobank	29,506	EDV, ESV, EF	Low
Ahlberg et al. (2021)^12^	LA	UK Biobank	35,658	Max volume, emptying fraction	Low
Khurshid et al. (2023)^22^	LV	UK Biobank	43,230	Mass, EDV, ESV	Low
Tcheandjieu et al. (2022)^8^	Aorta	UK Biobank	31,219	Diameter, distensibility	Low
Francis et al. (2022)^7^	Aorta	UK Biobank	32,590	Strain, peak velocity	Low
Aung et al. (2019)^23^	LV	UK Biobank	16,923	EDV, ESV, EF	Low
Pirruccello et al. (2020)^24^	LV	UK Biobank	36,041	EDV, ESV, SVi	Low
Magnani et al. (2014)^25^	LA	Framingham Heart Study Cohorts	8416	Max volume	Moderate
Fox et al. (2013)^26^	LV	Community based cohorts of African Americans	6765	Mass, wall thickness	Moderate
Yu et al. (2023)^27^	LV	UK Biobank	31,219	EDV, ESV, EF	Low
Meyer et al. (2020)^28^	LV	UK Biobank	18,096	EDV, ESV, trabeculations	Low
Gomes et al. (2024)^29^	Aorta	UK Biobank	37,653	Area, distensibility	Low
Aung et al. (2023)^9^	LV	UK Biobank	42,176	Wall thickness, mass	Low
Schmidt et al. (2023)^30^	LV, RV	UK Biobank	36,548	EDV, ESV, EF (both chambers)	Low
Thanaj et al. (2022)^31^	LA, LV	UK Biobank	39,559	LA volume, LV EF	Low
Pirruccello et al. (2023)^21^	Aorta	UK Biobank	42,342	Strain, systemic SV	Low

Abbreviations: LV = left ventricle; RV = right ventricle; LA = left atrium; RA = right atrium; EDV = end-diastolic volume; ESV = end-systolic volume; EF = ejection fraction; SV = stroke volume; SVi = stroke volume indexed.

Risk of bias assessment using PRISMA-modified criteria demonstrated high methodological quality across most studies. Fifteen studies were rated as low bias, employing population-based designs with standardised CMR protocols and appropriately adjusted GWAS analyses. Two studies (Magnani et al.^25^ and Fox et al.^26^) showed moderate bias due to their smaller sample sizes (<10,000 participants). This robust dataset enabled systematic identification of genetic variants associated with diverse cardiac traits across all chambers, with detailed chamber-specific findings presented in subsequent sections.

### Common genes across studies

**Fig. 2:**
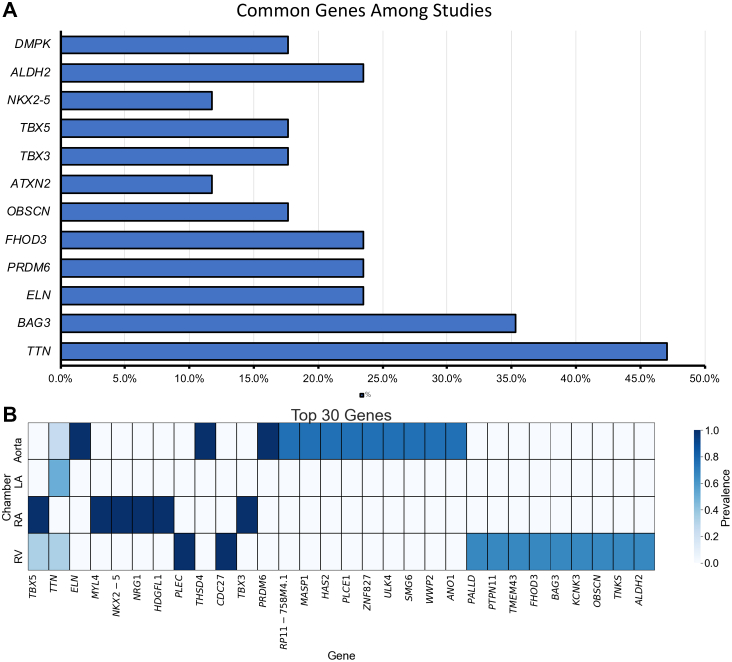
Gene associations with studies and cardiac traits. (A) Bar graph of prevalence of genes in the studied literature. (B) Heat map showing chamber-wise prevalence of top 30 common genes.

The most prevalent genes identified across all studies are illustrated in Fig. 2A. *TTN* appeared in highest number of studies (47.1%), followed by *BAG3* with 35.3% of the studies. Elastin (*ELN)* was found in 23.5% of the studies but mostly prevalent in aortic traits like diameter, area, forward peak velocity, strain, and distensibility. PR/SET domain 6 (*PRDM6), aldehyde dehydrogenase 2 (ALDH2)* and formin homology 2 domain containing 3 (*FHOD3)* were also prioritised in 23.5% of the studies, followed by obscurin *(OBSCN*), T-box transcription factor 5 *(TBX5),* dystrophia myotonica protein kinase *(DMPK) and* T-box transcription factor 3 *(TBX3)* with 17.6% each. Ataxin 2 *(ATXN2)* and NK2 homoeobox 5 (*NKX2-5)* were prevalent in 11.8% of the studies.

Fig. 2B shows the chamber-specific prevalence for each gene, out of the total studies identified for that chamber. Among the cardiac chambers, the LV had the highest number of studies which was 10, indicating strong research focus on LV-associated traits. However, the aorta exhibited the highest number of genes within the top 30, followed by the RV, suggesting a greater genetic diversity in their structural and functional associations. This highlights the proportional significance of these key genes in comparison to others within each cardiac chamber.

### Genes commonly associated with specific cardiac traits

In the LV, a total of 78 genes were shown to have associated with CMR based traits. Supplementary Figures S1 and S2 show a Manhattan–style plot of the genes, with the y-axis representing the number of studies, and x-axis representing the gene based on the chromosome-based location. *TTN* on chromosome 2 was associated with 15 LV traits, followed by *BAG3* associated with 14 traits. Similarly, genes associated with the RV include cell division cycle 27 (*CDC27)* on chromosome 17 with 8 associated traits, followed by *OBSCN* and *TTN* on chromosomes 1 and 2, respectively with 7 traits associated. Key genes influencing the aorta comprise of mostly *ELN* on chromosome 7, with 9 traits associated. Due to relatively low significant associations in studies for the LA and RA, *ANKRD1* on chromosome 10 had 3 associations with LA and *NKX2-5* (chromosome 5), *TBX3* (chromosome 12) and *MYL4* (chromosome 17) had association with 4 RA traits.

### Cross-chamber genetic associations in cardiac traits

We identified genes that could potentially affect mechanisms of multiple cardiac chambers in Table 2. Most genes had common associations among the left and right ventricles. Notable genes found for both LV and RV traits include protein tyrosine phosphatase nonreceptor type 11 (*PTPN11),* transmembrane protein 43 *(TMEM43), BAG3, TTN, FHOD3, TBX5, ATXN2, ALDH2* and *DMPK*. However, associations between other chambers also exist, with golgi SNAP receptor complex member 2 (*GOSR2)* gene implicated in both aorta and the RV, highlighting its potential role in cardiovascular structural integrity and disease variants.

**Table 2:** Key genes associated with multiple cardiac chambers, and their primary functions.

Gene	Chr	Cardiac chamber	Associated traits	Function
*TTN*	2	LV, RV	EDV, ESV, EF, SV, mass, trabeculations	Sarcomere assembly, myocardial elasticity
*TMEM43*	3	LV, RV	ESV, EF	Nuclear transcription, structural remodelling
*BAG3*	10	LV, RV	EDV, ESV, EF	Protein homoeostasis, myocyte contraction
*PTPN11*	12	LV, RV	EDV, ESV, SV	RAS/MAPK signalling, myocyte regulation
*TBX5*	12	LV, RV, RA	EDV (LV/RV), SV, RA volumes	Cardiac development, conduction system
*ATXN2*	12	LV, RV	EDV, SV	RNA metabolism, stress response
*ALDH2*	12	LV, RV	EDV, SV	Aldehyde detoxification, oxidative stress
*GOSR2*	17	RV, Aorta	EDV (RV), aortic strain	Vesicle trafficking, structural integrity
*FHOD3*	18	LV, RV	EF (LV), ESV (RV), wall thickness	Sarcomere organisation, actin polymerisation
*DMPK*	19	LV, RV	EDV, ESV	Calcium homoeostasis, muscle function

Abbreviations: Chr = chromosome; LV/RV/LA/RA = cardiac chambers; EDV/ESV/EF/SV = volumetric measures.

### Description of genes and their association with disease

We further explored the function of the key genes identified in our review and their association with cerebrovascular, autoimmune and broader medical diseases, in addition to CVDs (Table 3). The following section provides the gene function and possible disease associations for 15 key genes of interest we found in our review. A detailed version is provided in the Supplementary Material.

**Table 3:** Disease associations of cardiac genes: cardiovascular (CVD) and non-CVD conditions, with ClinGen based strength of association.

Gene	Disease association (ClinGen)	Strength of association	Other potential associations
*TTN*	DCM, *TTN*-related myopathy	Definitive	Heart failure, atrial fibrillation limb-girdle muscular dystrophy
*BAG3*	DCM, Myofibrillar myopathy	Definitive	HCM, arrhythmias, Parkinson's disease
*PTPN11*	Noonan syndrome	Definitive	HCM, pulmonary valve defects, juvenile myelomonocytic leukaemia
*TMEM43*	ARVC	Definitive	Conduction disorders
*FHOD3*	HCM	Definitive	DCM
*TBX5*	Holt-Oram syndrome	Definitive	ASD, AF
*ATXN2*	Spinocerebellar ataxia type 2	Definitive	CAD, heart failure, ALS
*DMPK*	–	N/A	DCM, conduction defects, myotonic dystrophy type 1
*ELN*	Cutis laxa	Definitive	Aortic aneurysms, supravalvular aortic stenosis
*TBX3*	–	N/A	AF, AV block, HCM
*NKX2-5*	ASD, VSD, conduction defects	Definitive	DCM
*GOSR2*	Progressive myoclonus epilepsy	Definitive	ARVC-like phenotypes
*MYL4*	–	N/A	Atrial fibrillation, congenital atrial defects

Abbreviations: DCM/HCM = dilated/hypertrophic cardiomyopathy; ARVC = arrhythmogenic RV cardiomyopathy; ASD/VSD = atrial/ventricular septal defect; AF = atrial fibrillation; CAD = coronary artery disease.

#### TTN

*TTN* encodes titin, a structural protein essential for sarcomere integrity and myocardial elasticity. It is one of the most frequently identified genes in cardiac GWAS, with associations to EDV, ESV and EF. *TTN* truncating variants are a major cause of dilated cardiomyopathy (DCM) and have been linked to AF and heart failure with preserved ejection fraction (HFpEF).^32^, ^33^, ^34^

#### BAG3

*BAG3* is crucial for protein homoeostasis and myocyte contraction, with variants linked to HFpEF and myofibrillar myopathy.^35^ Our findings associate *BAG3* with left ventricular EDV, ESV, and EF, potentially supporting its role in cardiac function and heart failure pathogenesis. *BAG3* deficiency impairs autophagy and protein quality control, leading to myocyte dysfunction and cardiomyopathy.^36^^,^^37^

#### ELN

*ELN* encodes elastin, a key extracellular matrix protein that maintains aortic elasticity. Variants are associated with supravalvular aortic stenosis and aortic aneurysms.^8^ Our findings link *ELN* to aortic diameter and distensibility, underscoring its importance in vascular integrity and risk of aortic dilation.^38^ However, these relationships could be confounded by undiagnosed aortic valve pathology and must be investigated further.

#### PRDM6

*PRDM6* regulates vascular smooth muscle cell differentiation, with variants linked to patent ductus arteriosus, CAD and AF.^7^^,^^39^

#### FHOD3

*FHOD3* is essential for sarcomere organisation and myocardial contractility. Variants are associated with hypertrophic cardiomyopathy (HCM) and DCM.^40^

#### OBSCN

*OBSCN* encodes obscurin, a cytoskeletal protein involved in sarcomere organisation and mechanical stability. Variants are linked to arrhythmogenic right ventricular cardiomyopathy (ARVC) and other cardiomyopathies.^41^

#### ATXN2

*ATXN2* is involved in RNA metabolism and cellular stress responses, with links to CAD, MI and HF.^42^

#### TBX3

*TBX3* is a transcription factor essential for sinoatrial and atrioventricular node differentiation. Variants disrupt cardiac conduction, leading to AV block, HCM, and HF.^43^

#### TBX5

*TBX5* is crucial for atrial and ventricular septation and conduction system development. Variants are associated with Holt-Oram syndrome, AF and Brugada syndrome.^44^

#### NKX2-5

*NKX2-5* is a key regulator of myocardial differentiation and septation, with variants showing association with atrial septal defect, tetralogy of Fallot, atrioventricular nodal reentrant tachycardia and AF.^45^^,^^46^

#### ALDH2

*ALDH2* encodes aldehyde dehydrogenase 2, which detoxifies aldehydes from alcohol metabolism and oxidative stress. Variants increase cardiovascular disease risk, including MI and alcohol-induced cardiac dysfunction.^47^

#### DMPK

*DMPK* is involved in muscle function and myotonic dystrophy type 1 (DM1). Variants lead to cardiac conduction defects, arrhythmias, and DCM, necessitating genetic screening in DM1 patients.^4^

#### PTPN11

*PTPN11* regulates cardiac development and Ras-MAPK signalling, with variants causing Noonan syndrome and HCM.^48^

#### TMEM43

*TMEM43* is involved in structural remodelling and nuclear transcription, with variants linked to ARVC.^49^^,^^50^

#### GOSR2

*GOSR2* encodes a Golgi SNARE protein involved in vesicle trafficking, with variants linked to progressive myoclonus epilepsy and cardiovascular disease risk.^51^^,^^52^

#### MYL4

*MYL4* encodes myosin light chain 4, essential for atrial contraction. Variants are linked to AF and congenital atrial defects.^53^

#### ANKRD1

*ANKRD1* regulates cardiac gene expression in response to mechanical stress. Variants are associated with DCM and HCM.^54^

## Discussion

This systematic review provides a comprehensive overview of genetic associations with cardiac structure and function derived from CMR-based GWAS. By analysing 17 studies, we identified 149 risk loci associated with CMR-derived traits across different cardiac chambers. Prominent genes such as *TTN, BAG3*, *PTPN11*, and *TMEM43* were found to be associated with both left and right ventricular traits, primarily involved in structural roles and cell signalling pathways.^37^^,^^48^^,^^49^ Additionally, genes like *ELN* were specifically linked to aortic traits, highlighting their role in aortic elasticity and function.^38^ The integration of GWAS data with CMR traits has provided a detailed genetic map linking these traits to CVDs such as DCM and HF. These findings underscore the interconnectedness of cardiovascular health with cerebrovascular and metabolic diseases, emphasising the pleiotropic effects of these genetic markers.

The genetic variants identified in this study may inform personalised cardiovascular medicine, although further studies are warranted to confirm their clinical relevance. *TTN* truncating variants, recognised as a major cause of DCM,^55^ and *BAG3* variants, linked to both heart failure and myofibrillar myopathy,^35^^,^^36^ may represent potential targets for early screening and intervention in high-risk individuals, although further studies are needed to confirm their clinical utility. Similarly, *ELN* variants associated with aortic aneurysms could transform surveillance strategies for aortopathies.^38^^,^^56^ The incorporation of these variants into polygenic risk scores, as demonstrated in recent large-scale studies,^57^^,^^58^ may enhance risk stratification and enable proactive management of cardiovascular diseases, although further research is needed to substantiate these findings. Beyond diagnostics, mechanistic insights into genes such as *PRDM6* and *FHOD3* reveal novel therapeutic opportunities. *PRDM6*'s role in vascular smooth muscle differentiation positions it as a potential target for vascular remodelling therapies,^39^ while *FHOD3*'s regulation of sarcomere organisation offers a pathway to improve contractility in HCM.^59^ These findings align with emerging precision cardiology frameworks and underscore the potential of genetics to guide tailored interventions across the cardiovascular disease spectrum.^60^

**Fig. 3:**
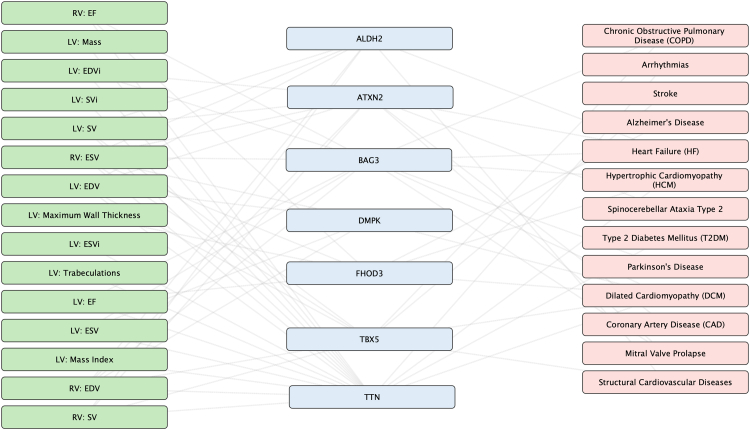
Schematic diagram of trait-gene-disease association for the most associated genes across the left and right ventricles.

Fig. 3 illustrates significant genetic associations between cardiac structural/functional traits and a spectrum of diseases, revealing both expected and novel biological links. On the cardiac trait side (left), GWAS analyses identified key volumetric measures (LV/RV EDV, ESV, SV, EF) and structural parameters (mass, wall thickness, trabeculations) associated with specific genes, including well-known cardiac regulators like *TTN* (sarcomere function) and *BAG3* (protein quality control), as well as metabolic genes like *ALDH2*. The disease associations (right) demonstrate these genes' pleiotropic effects, with cardiovascular conditions (DCM, HCM, CAD) being most prevalent, but also extending to neurological (Alzheimer's, Parkinson's, spinocerebellar ataxia) and metabolic disorders like Type 2 Diabetes Mellitus. These findings underscore the complex interplay between cardiac biology and systemic disease, highlighting how genes maintaining cardiac structure/function may also influence broader physiological systems through shared molecular pathways; further mechanistic studies are warranted to support this hypothesis.

Studies like Vad et al. show that CMR-derived traits can help predict outcomes such as cardioembolic stroke, and genes with pleiotropic effects may strengthen multi-trait PRS models.^61^ The genetic variants identified in this review could theoretically inform polygenic risk score (PRS) development, yet recent large-scale evaluations urge caution in the use of polygenic risk scores in screening and prediction. Hingorani et al. highlight significant constraints in their analysis of nearly 1000 PRSs, revealing weak performance with detection rates of only 11% for a 5% false positive rate for a range of conditions e.g., coronary artery disease.^62^ Additionally, our findings highlight additional challenges due to: (1) ancestry-specific effects, given the European bias in current CMR GWAS, and (2) integration of CMR traits with clinical risk factors.

Recent studies have identified genetic loci associated with various valvular heart diseases, highlighting the genetic underpinnings of valve function. For instance, a GWAS meta-analysis involving over 900,000 participants identified 32 loci associated with calcific aortic valve stenosis (CAVS), with several genes exhibiting tissue-specific expression in aortic valve tissues.^63^ Additionally, a transcriptome-wide association study (TWAS) found that PALMD expression is causally associated with CAVS risk.^64^ These findings underscore the importance of genetic factors in valve function and disease. While our study primarily focused on chamber volumes and structure, several genes identified in our review—such as *ELN*, *PTPN11* and *TBX5* have known roles in cardiovascular development and structural integrity, suggesting they may also contribute to valve formation and function. Integrating valve-specific phenotypes in future analyses could therefore provide a more comprehensive understanding of cardiac morphology and its genetic determinants.

While this review provides a robust genetic map of cardiac traits, several limitations must be acknowledged. The predominance of European-ancestry populations in included studies may limit generalisability to other ethnic groups. Additionally, methodological heterogeneity across studies in phenotyping protocols and analytical approaches may affect result comparability. While our inclusive approach captured all reported genetic associations with CMR traits, some studies utilised overlapping cohorts (e.g., UK Biobank), which may inflate the apparent prevalence of certain genetic signals. However, this strategy allowed us to document the full spectrum of associations in the literature. Subsequently, as CMR most accurately characterises LV structure and function, our review was disproportionately focused on LV traits, which may skew the observed gene associations towards those relevant to the LV, which remains a significant limitation. Our results could also be further strengthened by incorporating summary statistic-based genetic correlations with cardiovascular diseases. Additionally, Mendelian Randomisation would offer causal inference and improve the interpretability of these associations.

These limitations highlight the need for more diverse cohorts and standardised methodologies. Future studies should prioritise several key directions: First, expanding recruitment to underrepresented populations will ensure more equitable applicability of genetic findings. Second, longitudinal studies are needed to elucidate how genetic predispositions interact with environmental factors over time. Third, integrating multi-omics approaches will provide deeper mechanistic insights into gene function. Finally, translating these genetic discoveries into clinical applications will require developing and validating polygenic risk scores and targeted therapies.

In conclusion, this systematic review provides a comprehensive atlas of genetic associations with cardiac structure and function, as derived from CMR-based GWAS. By identifying key genetic variants and their associations with cardiac traits, this review enhances our understanding of the genetic architecture underlying cardiovascular diseases and other medical conditions. These findings provide a foundation for future studies to advance personalised medicine, risk stratification, and therapeutic development toward more precise and effective management of cardiovascular diseases. Future research should focus on functional validation, multi-omics integration, and the inclusion of diverse populations to further advance this field and improve cardiovascular outcomes globally.

## Contributors

SL was responsible for data curation, formal analysis, investigation, and drafting of the manuscript. RHD contributed to reviewing and editing the manuscript and validation of the analyses. ADH was involved in funding acquisition, reviewing and editing the manuscript, and validation. NP led the conceptualisation and methodology, supervised the project, and contributed to reviewing and editing the manuscript. All authors have read and approved the final version of the manuscript.

## Data sharing statement

The datasets used and analysed during this systematic review are available from the corresponding publications included in the study. No new primary data were generated.

## Declaration of interests

All authors declare no relevant conflict of interest.

## Appendix A Supplementary data

Supplementary Material

Gene Prioritisation
