# Supplementary material for: A systematic review of GWAS on CMR imaging traits: genetic insights into cardiovascular structure, function, and diseases
Source: eBioMedicine. 2025 Oct 30;121:105992. doi: 10.1016/j.ebiom.2025.105992 (PMC12616015; doi:10.1016/j.ebiom.2025.105992)
Supplement: Supplementary Material [file mmc1.docx]

**Supplementary Material**

***TTN***

The *TTN* gene is essential for sarcomere assembly and myocardial passive stiffness. *TTN* encodes titin, a giant protein that spans half of the sarcomere from the Z-disk to the M-line and plays a crucial role in maintaining the structural integrity and elasticity of the myocardium. Previous studies have identified *TTN* as a significant gene associated with various cardiac traits such as end-diastolic volume (EDV), end-systolic volume (ESV), and ejection fraction (EF) [1]. Mutations have been linked to conditions such as dilated cardiomyopathy (DCM) and HCM, atrial fibrillation and sick sinus syndrome [1,2]. Our findings convey the critical role of *TTN*, which appeared in 44% of the studies analyzed, indicating its significant impact on cardiac structure and function. The gene's large size, encompassing 363 exons, and the high frequency of its mutations underscore its pivotal role in myocardial function [2]. A study by Herman et al. (2012) demonstrated that truncating variants in *TTN* (*TTN*tv) are particularly prevalent in patients with idiopathic DCM, accounting for up to 25% of familial and 18% of sporadic cases [3]. *TTN*tv lead to a truncated, non-functional titin protein, which compromises the sarcomere's structural integrity and elasticity, thereby impairing myocardial function. This impairment results in ventricular dilation and systolic dysfunction, characteristic features of DCM [3]. Further elucidation of *TTN*'s role in DCM was provided by Roberts et al. (2015), who performed an integrated analysis of allelic, transcriptional, and phenotypic data [4]. Their study highlighted that *TTN* truncations significantly disrupt sarcomere function, leading to a cascade of maladaptive myocardial remodeling and heart failure [4]. These findings may emphasize the clinical relevance of *TTN* mutations in the pathogenesis of DCM and the importance of genetic screening for *TTN* variants in patients presenting with idiopathic DCM. They also demonstrated that *TTN*tv carriers exhibit a broad range of cardiac phenotypes, from asymptomatic individuals to those with severe heart failure requiring heart transplantation. The study also highlighted the variable penetrance and expressivity of *TTN* mutations, suggesting that additional genetic, epigenetic, or environmental factors could modulate the clinical manifestations of *TTN*-related cardiomyopathy [4]. Clinically, the identification of *TTN* mutations has profound implications for patient management. Routine genetic screening for *TTN* mutations in patients with idiopathic DCM has the potential to facilitate early diagnosis and intervention, potentially improving clinical outcomes. Moreover, *TTN* mutation carriers may benefit from tailored therapeutic strategies, including close monitoring for the development of heart failure, lifestyle modifications, and pharmacological interventions aimed at preserving cardiac function [4].

***BAG3***

The *BAG3* gene is involved in myocyte structure and contraction. *BAG3* encodes a co-chaperone protein that interacts with the Hsp70 chaperone system, playing a critical role in maintaining protein homeostasis and cellular stress responses. Mutations in *BAG3* have been linked to heart failure and myofibrillar myopathy [5]. Previous research has shown that *BAG3* mutations could lead to dilated cardiomyopathy (DCM) and various forms of heart failure [5]. Our study supports these findings, identifying *BAG3* in 28% of the total studies and associating it with end-diastolic volume (EDV), end-systolic volume (ESV), and ejection fraction (EF), further emphasizing its potential significance in maintaining cardiac function. *BAG3* mutations disrupt autophagy and protein homeostasis, leading to myofibrillar disarray and impaired cardiac function [5]. A comprehensive review by Knezevic et al. (2015) highlights the crucial role of *BAG3* in the heart failure paradigm [6]. The study underscores that *BAG3* mutations contribute to the pathogenesis of heart failure by impairing the autophagy-lysosome pathway and disrupting the degradation of misfolded proteins [6]. This disruption leads to the accumulation of damaged proteins and organelles, contributing to myofibrillar disarray and cardiomyocyte death. These pathological changes manifest as dilated cardiomyopathy and heart failure [6]. Further research has shown that *BAG3* is essential for the mechanical stability and contractility of cardiomyocytes. *BAG3*-deficient mice exhibit rapid development of cardiomyopathy and early death, indicating the gene's vital role in cardiac function [6]. In humans, *BAG3* mutations have been associated with a spectrum of cardiomyopathies, from mild forms to severe heart failure requiring cardiac transplantation [5,6]. The clinical relevance of *BAG3* mutations extends beyond structural heart disease. *BAG3* has also been linked to the modulation of inflammatory responses and apoptosis, processes that are critical in the progression of heart failure [6]. This multifaceted role of *BAG3* makes it a potential therapeutic target. Interventions aimed at enhancing *BAG3* function or mimicking its activity could offer new avenues for the treatment of heart failure and other related conditions. Recent studies have also identified an association between *BAG3* mutations and non-cardiac diseases such as Parkinson's disease and chronic obstructive pulmonary disease (COPD). A study by Ying et al. (2022) demonstrated that *BAG3* is involved in the maintenance of cellular homeostasis in neurons, with mutations contributing to the development of Parkinson's disease through mechanisms related to protein aggregation and neuronal apoptosis [7]. Additionally, *BAG3* mutations have been implicated in COPD, where impaired autophagy and protein homeostasis lead to chronic inflammatory responses and lung tissue damage [8]. These findings highlight the broader impact of *BAG3* beyond cardiac function, emphasizing its potential role in maintaining cellular integrity across multiple organ systems.

***ELN***

The *ELN* gene is crucial for maintaining the elasticity of the aorta. *ELN* encodes elastin, a key extracellular matrix protein that provides resilience and elastic recoil in the aorta and other large arteries. Mutations in *ELN* can result in conditions such as supravalvular aortic stenosis and ascending aortic aneurysms [9]. Previous studies have highlighted the role of *ELN* in aortic health, demonstrating its critical function in vascular integrity and elasticity [9]. Our review affirms these findings, showing *ELN*'s possible association with aortic diameter and distensibility in 22% of the studies analysed. Mutations in *ELN* have shown to decrease elasticity and increased susceptibility to aortic dilation and aneurysms. The pathology associated with *ELN* mutations includes supravalvular aortic stenosis, a condition characterized by the narrowing of the ascending aorta, which can lead to obstructed blood flow and increased cardiac workload. Furthermore, *ELN* mutations are implicated in ascending aortic aneurysms, where the weakened aortic wall leads to abnormal dilation and a risk of rupture [9]. A study by Milewicz et al. (2010) provided significant insights into the clinical manifestations and genetic underpinnings of *ELN*-related vascular diseases [10]. The study showed that individuals with *ELN* mutations often present with multiple vascular anomalies, including arterial tortuosity and skin laxity, which are indicative of generalized elastin deficiency [10]. These findings emphasize the systemic nature of *ELN*-related pathologies, extending beyond the aorta to other vascular and connective tissues. The clinical relevance of *ELN* mutations may extend to their role in elastin insufficiency associated with aortic and pulmonary diseases. Elastin is critical for the proper function of the aortic and pulmonary valves, and its deficiency can lead to valve insufficiency and associated complications. Additionally, elastin excess associated with ascending aortic dilation can lead to ascending aortic aneurysm, patent ductus arteriosus (PDA), and atrial septal defect (ASD) [10]. Patients with *ELN* mutations may present with a variety of cardiovascular manifestations, necessitating comprehensive cardiovascular assessment and long-term monitoring. Genetic screening for *ELN* mutations in individuals with a family history of aortic aneurysms and related conditions can facilitate early diagnosis and preventive care.

***PRDM6***

The *PRDM6* gene is important for smooth muscle cell differentiation and embryonic development. *PRDM6* encodes a transcriptional repressor that plays a critical role in the differentiation of vascular smooth muscle cells. Mutations in *PRDM6* have been linked to familial patent ductus arteriosus (PDA) and increased risks of coronary artery disease (CAD) and AF [11]. Our study supports these associations, identifying *PRDM6* as a key gene involved in cardiovascular development and disease, consistent with prior research. *PRDM6* mutations disrupt normal vascular development and contribute to the pathology of PDA and other cardiovascular anomalies. A study by Li et al. (2016) highlighted the role of *PRDM6* in the pathogenesis of familial PDA, showing that mutations in *PRDM6* impair the differentiation of vascular smooth muscle cells, leading to the persistence of the ductus arteriosus after birth [12]. This impairment results in abnormal blood flow between the aorta and pulmonary artery, which can lead to heart failure if untreated. Further research by Yu et al. (2023c) demonstrated that *PRDM6* is involved in regulating the expression of genes essential for smooth muscle cell contractility and extracellular matrix composition [13]. This regulation is crucial for maintaining vascular integrity and function. Mutations in *PRDM6* disrupt these processes, leading to vascular anomalies such as CAD and AF [13]. The study emphasized that *PRDM6* mutations contribute to a pro-inflammatory vascular environment, which accelerates the development of atherosclerosis and atrial arrhythmogenesis. Clinical relevance of *PRDM6* mutations extends to their impact on body mass index (BMI) and breast cancer. *PRDM6* has been linked to variations in BMI, indicating a potential role in metabolic regulation and obesity-related cardiovascular risks [13]. Additionally, *PRDM6* has been implicated in breast cancer, suggesting that its regulatory functions in cellular differentiation and proliferation may extend to oncogenesis.

***FHOD3***

The *FHOD3* gene regulates sarcomere organization in cardiomyocytes. *FHOD3* encodes a formin protein that modulates actin filament dynamics, essential for sarcomere stability and myocardial contractility. Mutations in *FHOD3* are associated with HCM and dilated cardiomyopathy (DCM) [14]. Our review found *FHOD3* to be potentially associated with various cardiac traits, supporting previous findings of its role in maintaining cardiac structure and function [14]. A study by Ochoa et al. (2018) demonstrated that *FHOD3* mutations result in altered actin filament dynamics, which directly impacts the structural integrity and function of cardiac muscle fibers, leading to compromised contractility [15]. The study further emphasized that these mutations lead to sarcomere disarray and impaired force generation in cardiomyocytes, which are hallmark features of HCM and DCM [15]. Moreover, *FHOD3* has been implicated in neurodegenerative conditions such as Alzheimer's disease and vascular conditions like varicose veins. This broader implication highlights the gene's role beyond cardiac health, affecting other organ systems where actin dynamics are crucial [16]. Clinically, identifying *FHOD3* mutations can aid in the early diagnosis and management of cardiomyopathies. Genetic screening for *FHOD3* variants in patients with unexplained HCM or DCM has been shown to facilitate timely interventions and personalized treatment plans. Additionally, understanding the pathways affected by *FHOD3* mutations may provide insights into novel therapeutic targets that address the underlying molecular dysfunctions. These findings underscore the importance of *FHOD3* in both the structural and functional aspects of the heart.

***OBSCN***

The *OBSCN* gene is involved in cytoskeletal structure and regulation. *OBSCN* encodes obscurin, a giant protein that connects the sarcomere to other cellular components, ensuring proper alignment and mechanical stability within the muscle cells. Mutations in *OBSCN* are linked to arrhythmogenic right ventricular cardiomyopathy (ARVC) and other cardiomyopathies [17]. Our findings may indicate that *OBSCN* plays a role in both right and left ventricular function, corroborating previous research on its involvement in cardiac diseases [17]. Mutations in *OBSCN* can disrupt sarcomeric alignment and mechanical stability, contributing to the development of cardiomyopathies. A study by Ye et al. (2019) reevaluated genetic variants previously associated with ARVC by integrating population-based cohorts and proteomics data. The study highlighted that certain *OBSCN* variants significantly contribute to the pathogenesis of ARVC by affecting the structural integrity and function of cardiac muscle cells [18]. This disruption leads to arrhythmias and increased susceptibility to sudden cardiac death. Further elucidation of *OBSCN*'s role in cardiac disease was provided by Grogan and Kontrogianni-Konstantopoulos (2019), who reviewed the mechanisms through which obscurin influences cardiac health [19]. They emphasized that obscurin's interactions with other sarcomeric proteins are vital for maintaining the structural integrity of cardiomyocytes. Disruption of these interactions due to *OBSCN* mutations can lead to mechanical instability and cardiomyopathy progression [19]. Additionally, Marston (2017) explored the impact of obscurin variants on inherited cardiomyopathies [20]. The study found that *OBSCN* mutations are associated with a spectrum of cardiac phenotypes, including HCM and dilated cardiomyopathy (DCM). These mutations can cause defects in sarcomere assembly and function, leading to impaired cardiac contractility and heart failure [20]. Clinically, the identification of *OBSCN* mutations is crucial for the early diagnosis and management of cardiomyopathies. Genetic screening for *OBSCN* variants in patients with unexplained cardiac symptoms or a family history of cardiomyopathy can facilitate timely interventions and personalized treatment plans. Understanding the specific pathways disrupted by *OBSCN* mutations can also aid in developing targeted therapies that address the underlying molecular dysfunctions.

***ATXN2***

The *ATXN2* gene has been linked to multiple cardiovascular traits and diseases such as coronary artery disease (CAD), myocardial infarction (MI), and HF [21]. Our study identified *ATXN2* in association with numerous cardiac traits, highlighting its broad impact on cardiovascular health, consistent with existing literature [21]. *ATXN2* influences RNA metabolism and cellular stress responses. Mutations in *ATXN2* have been associated with neurodegenerative disorders and cardiovascular diseases, suggesting a shared molecular pathway that impacts both neuronal and cardiac tissues [21]. A study by van der Ende et al. (2018) revealed that *ATXN2* variants are associated with altered cardiac function and structure, contributing to the development of heart failure [22]. Further research by Dávalos-Rodríguez et al. (2022) investigated the VNTR (CAG)n polymorphism of the *ATXN2* gene and its association with metabolic parameters of cardiovascular risk in the Amerindian population of Oaxaca [23]. The study found that certain polymorphisms in *ATXN2* are linked to increased cardiovascular risk factors, such as obesity and dyslipidemia, which are known contributors to coronary artery disease (CAD) and MI [23]. This research underscores the gene's involvement in metabolic regulation and its broader implications for cardiovascular health. Additionally, *ATXN2* has been implicated in neurodegenerative disorders, such as spinocerebellar ataxia type 2 (SCA2) and amyotrophic lateral sclerosis (ALS) [24]. The shared molecular pathways between neurodegenerative and cardiovascular diseases suggest that *ATXN2* mutations can have systemic effects, impacting both neuronal and cardiac tissues. Clinically, the identification of *ATXN2* mutations is essential for understanding the genetic basis of complex cardiovascular and neurodegenerative diseases. Genetic screening for *ATXN2* variants can aid in early diagnosis and risk assessment for conditions such as CAD, MI, and HF. Moreover, understanding the pathways affected by *ATXN2* mutations may provide insights into novel therapeutic targets that address both cardiovascular and neurodegenerative aspects of the disease [24].

***TBX3***

The *TBX3* gene plays a crucial role in atrioventricular (AV) conduction and the differentiation of the sinoatrial (SA) and atrioventricular (AV) nodes. *TBX3* is a transcription factor involved in the development of the cardiac conduction system, essential for maintaining normal heart rhythm. Variants in *TBX3* lead to AV block, HCM, coronary artery disease (CAD), and HF [25]. Our review may confirm the significant role of *TBX3* in cardiac function and its association with various cardiac traits, supporting previous studies [25]. *TBX3* mutations can cause conduction defects and structural abnormalities in the heart, leading to various cardiac pathologies. Delisle et al. (2019) explored the role of *TBX3* in the development and function of the cardiac conduction system, highlighting that *TBX3* mutations disrupt normal RNA processing and alternative splicing, which are crucial for the proper development of the SA and AV nodes [26]. This disruption can result in AV block, where the electrical signal between the atria and ventricles is impaired, leading to bradycardia and other arrhythmias [26]. Further research by Bakker et al. (2008) has shown that *TBX3* is involved in the regulation of several genes critical for cardiac development and function [27]. Mutations in *TBX3* have been associated with structural heart defects, including atrial and ventricular septal defects, which can compromise cardiac output and lead to heart failure [27]. Additionally, *TBX3* has been implicated in the pathogenesis of HCM, a condition characterized by thickened ventricular walls and impaired cardiac function [28]. The clinical relevance of *TBX3* extends beyond cardiac conduction and structural defects. *TBX3* mutations have also been linked to ischemic stroke, Alzheimer's disease, colorectal cancer, breast cancer, endometrial cancer, benign prostatic hyperplasia (BPH), chronic obstructive pulmonary disease (COPD), and hypertension (HTN) [29]. This broad spectrum of associated conditions underscores the gene's importance in various physiological processes and its potential impact on multiple organ systems.

***TBX5***

The *TBX5* gene is associated with early cardiac development and transcriptional activation of genes linked to cardiomyocyte maturation. *TBX5* is essential for the formation of the atrial and ventricular septa and the development of the conduction system. Mutations in *TBX5* can result in Holt-Oram syndrome, AF, Brugada syndrome, and dilated cardiomyopathy (DCM) [25]. Our findings support the role of *TBX5* in cardiac development and function, as reported in prior research [25]. *TBX5* plays a pivotal role in the formation of the heart's structural and functional components. Steimle and Moskowitz (2017) reviewed *TBX5*'s functions, highlighting its importance in the development of the cardiac septa and conduction system [30]. They emphasized that *TBX5* mutations can lead to significant congenital heart defects, including atrial and ventricular septal defects, which disrupt normal cardiac physiology and can result in heart failure [30]. Van Ouwerkerk et al. (2022) further investigated the impact of *TBX5* mutations by examining the *TBX5*-G125R variant in patient-specific models [31]. Their study demonstrated that this variant induces profound transcriptional deregulation, leading to atrial dysfunction and contributing to the pathogenesis of AF and other arrhythmias. The research underscores the gene's role in maintaining normal atrial function and highlights the severe consequences of *TBX5* mutations on cardiac rhythm [31]. *TBX5*'s involvement in Brugada syndrome and DCM further illustrates its critical role in cardiac health. Brugada syndrome is characterized by a distinct electrocardiogram pattern and an increased risk of sudden cardiac death, often linked to defects in the cardiac conduction system and arrythmias [28]. Moreover, *TBX5* mutations are linked to cardioembolic stroke, venous thromboembolism (VTE), prostate cancer, breast cancer, and chronic obstructive pulmonary disease (COPD) [32]. This wide range of associated conditions indicates *TBX5*'s systemic influence, affecting not only cardiac development but also other physiological processes.

***NKX2***

The *NKX2-5* gene is critical for myocardial regeneration and differentiation of cardiac precursor cells. *NKX2-5* is a homeobox gene that regulates the expression of several cardiac-specific genes. Mutations in *NKX2-5* may be linked to familial atrial septal defect (ASD), tetralogy of Fallot (TOF), and AF [32]. Our study highlights the importance of *NKX2-5* in cardiac development and its potential association with various cardiac traits, consistent with existing literature [32]. *NKX2-5* mutations disrupt normal cardiac development, leading to congenital heart defects and arrhythmias. Cao et al. (2023) emphasized *NKX2-5*'s crucial role in cardiac development, regeneration, and diseases [33]. They demonstrated that *NKX2-5* mutations impair the formation of the cardiac septa and conduction system, which are essential for adequate cardiac function. This impairment can lead to a range of congenital heart defects such as ASD and TOF, as well as arrhythmias including AF [33]. Further research has shown that *NKX2-5* is involved in the regulation of myocardial cell proliferation and differentiation. Mutations in this gene affect the normal proliferation of cardiac precursor cells, leading to defects in heart formation and function. The study by Cao et al. (2023) also highlighted that *NKX2-5* plays a role in myocardial regeneration, suggesting that it could be a target for therapeutic interventions aimed at promoting heart repair and regeneration after injury [33]. Additionally, *NKX2-5* has been implicated in cardioembolic stroke, a condition characterized by the formation of blood clots in the heart that can travel to the brain and cause an ischemic stroke. This association emphasises the gene's importance in maintaining normal cardiac rhythm and preventing thromboembolic events [34]. Clinically, the identification of *NKX2-5* mutations is essential for the early diagnosis and management of congenital heart defects and arrhythmias. Genetic screening for *NKX2-5* variants in patients with a family history of congenital heart defects or unexplained arrhythmias can facilitate timely interventions and personalized treatment plans. Understanding the pathways affected by *NKX2-5* mutations may provide insights into novel therapeutic targets that address both structural and functional cardiac abnormalities [32,33].

***ALDH2***

The *ALDH2* gene is involved in the detoxification of aldehydes, which are toxic by-products of alcohol metabolism and lipid peroxidation. *ALDH2* encodes the enzyme aldehyde dehydrogenase 2, which is crucial for converting acetaldehyde, a toxic metabolite, into acetate, a less harmful substance. Mutations in *ALDH2* can result in altered aldehyde metabolism, leading to an increased risk of cardiovascular diseases such as myocardial infarction (MI) and alcohol-induced cardiac conditions [35]. Identifying individuals with *ALDH2* mutations can aid in the development of personalized treatment plans that include lifestyle modifications and pharmacological interventions to mitigate cardiovascular risks [36]. Zhang et al. (2023) provided a comprehensive review of the role of *ALDH2* in cardiovascular disease. The study emphasized that *ALDH2* deficiency, often due to the *ALDH2**2 variant, leads to an accumulation of acetaldehyde, which can cause oxidative stress, endothelial dysfunction, and inflammation. These pathological processes contribute to the development and progression of cardiovascular diseases, including myocardial infarction and heart failure [36]. The clinical implications of *ALDH2* mutations are significant. For example, individuals carrying the *ALDH2**2 allele are at a higher risk of developing alcohol-induced cardiac conditions due to impaired acetaldehyde detoxification. This risk is compounded by lifestyle factors such as alcohol consumption, which can exacerbate the adverse effects of *ALDH2* deficiency. Personalized treatment plans for these individuals should include recommendations for reduced alcohol intake and potential use of antioxidants to mitigate oxidative stress [36,37]. Furthermore, *ALDH2* may play a protective role against age-related cardiovascular diseases. Studies have shown that *ALDH2* activity decreases with age, which may contribute to the increased incidence of cardiovascular diseases in older adults. Enhancing *ALDH2* activity through pharmacological agents or gene therapy could offer new therapeutic avenues for preventing and treating cardiovascular conditions associated with *ALDH2* deficiency [36].

***DMPK***

The *DMPK* gene is primarily known for its role in myotonic dystrophy type 1 (DM1), a condition that affects both skeletal and cardiac muscles. *DMPK* encodes the myotonic dystrophy protein kinase, which is involved in muscle function and maintenance. Mutations in *DMPK* can lead to conduction defects, arrhythmias, and dilated cardiomyopathy (DCM) [38]. Genetic testing for *DMPK* can facilitate early diagnosis and intervention, improving management strategies for patients with myotonic dystrophy and associated cardiac complications [38]. McBride et al. (2022) reviewed the cardiac involvement and arrhythmias associated with myotonic dystrophy, highlighting that *DMPK* mutations disrupt normal muscle function, leading to various cardiac abnormalities [39]. The study emphasized that conduction defects, such as atrioventricular (AV) block and bundle branch block, are common in patients with DM1. These conduction defects can progress to more severe arrhythmias, including AF and ventricular tachycardia (VT), increasing the risk of sudden cardiac death [39]. Similarly, research by Ueda et al. (2000) explored the molecular mechanisms through which *DMPK* mutations cause cardiac complications [40]. Their study demonstrated that the expanded CTG repeats in the *DMPK* gene result in toxic RNA transcripts that sequester RNA-binding proteins. This sequestration disrupts the normal splicing of several cardiac genes, leading to abnormalities in cardiac muscle function and structure [40]. The accumulation of toxic RNA and the resultant protein misfolding are key pathological features of DM1, contributing to the development of cardiomyopathy and arrhythmias [40]. The clinical implications of *DMPK* mutations extend to the management of myotonic dystrophy. Identifying individuals with *DMPK* mutations through genetic testing allows for early intervention and personalized treatment plans. These plans may include regular cardiac monitoring, the use of pacemakers or implantable cardioverter-defibrillators (ICDs) to prevent sudden cardiac death, and pharmacological treatments to manage arrhythmias [38,39].

***PTPN11***

The *PTPN11* gene regulates cell signalling pathways and myocyte function. *PTPN11* encodes the protein tyrosine phosphatase SHP-2, which is involved in several signalling pathways crucial for cardiac development and function. Genetic variation in *PTPN11* has been associated with Noonan syndrome and LEOPARD syndrome, both of which can lead to structural heart defects and HCM [41]. Our review established that *PTPN11* may be frequently associated with multiple cardiovascular traits, corroborating its significant role in cardiac health as reported in previous studies [41]. *PTPN11* mutations lead to gain-of-function changes in the SHP-2 protein, resulting in enhanced signalling through the Ras-MAPK pathway. This aberrant signalling disrupts normal cardiac development and function, contributing to congenital heart defects and hypertrophic cardiomyopathy. Lauriol and Kontaridis (2011) reviewed the impact of *PTPN11*-associated mutations on cardiac health, emphasizing that these mutations are a major cause of the structural abnormalities observed in Noonan syndrome and LEOPARD syndrome [42]. They highlighted that the hyperactivation of the Ras-MAPK pathway due to SHP-2 gain-of-function mutations leads to excessive cell proliferation and abnormal cardiac tissue remodelling, which are key features of these syndromes [42]. Further research has shown that *PTPN11* mutations are also associated with peripheral arterial disease and hypertension. The involvement of SHP-2 in vascular smooth muscle cell function and blood vessel formation suggests that *PTPN11* mutations may contribute to vascular diseases by disrupting normal vascular development and function [43]. This broader impact on the cardiovascular system defines the gene's critical role in maintaining vascular health. Clinical studies have demonstrated that patients with *PTPN11* mutations often present with a variety of cardiovascular anomalies, including atrial septal defects (ASD), ventricular septal defects (VSD), pulmonary valve stenosis, and hypertrophic cardiomyopathy. The identification of *PTPN11* mutations in patients with these conditions can facilitate early diagnosis and intervention, improving management strategies and clinical outcomes [43,44].

***TMEM43***

Known for its role in structural remodelling and nuclear transcription, *TMEM43* mutations are linked to arrhythmogenic right ventricular cardiomyopathy (ARVC), a condition that can lead to sudden cardiac death [45]. Our findings are consistent with existing literature, identifying *TMEM43* in association with various cardiac traits, highlighting its involvement in both right and left ventricular function [45]. *TMEM43* variants result in defective intercalated discs, which are crucial for mechanical and electrical coupling between cardiomyocytes. This underscores the importance of structural integrity in preventing arrhythmogenic conditions and sudden cardiac death. A study by Merner et al. (2008) identified a missense mutation in the *TMEM43* gene as the cause of arrhythmogenic right ventricular cardiomyopathy type 5 (ARVC5), a fully penetrant, lethal arrhythmic disorder. The study demonstrated that individuals with this mutation exhibit severe structural and functional cardiac abnormalities, leading to ventricular arrhythmias and an increased risk of sudden cardiac death [46]. The *TMEM43* mutation disrupts the normal function of intercalated discs, impairing the mechanical and electrical connections between cardiomyocytes, which are essential for synchronized cardiac contraction [46]. Further research by Zink et al. (2022) explored the impact of altered *TMEM43* expression on cardiac structure and function using zebrafish models [47]. The study showed that reduced expression of *TMEM43* led to abnormal cardiac morphology and impaired contractility. These findings highlight the gene's crucial role in maintaining cardiac structural integrity and function [47]. The zebrafish model provided insights into the developmental and functional consequences of *TMEM43* mutations, reinforcing the gene's significance in cardiac health. *TMEM43*'s involvement in both right and left ventricular function suggests that its mutations can have widespread effects on the heart. The defective intercalated discs resulting from *TMEM43* mutations compromise the mechanical stability and electrical conductivity of the myocardium, making the heart more susceptible to arrhythmias and mechanical failure [45]. This broad impact highlights the importance of structural integrity in preventing arrhythmogenic conditions and maintaining overall cardiac function. Clinically, identifying *TMEM43* mutations is crucial for the early diagnosis and management of ARVC and related cardiomyopathies. Genetic screening for *TMEM43* variants in patients with a family history of sudden cardiac death or unexplained arrhythmias can facilitate timely interventions and personalized treatment plans. Understanding the pathways affected by *TMEM43* mutations can also aid in developing targeted therapies that address the underlying structural defects.

***GOSR2***

Mutations in *GOSR2* have been associated with progressive myoclonus epilepsy and certain neurodegenerative conditions [48,49]. *GOSR2* encodes the Golgi SNAP receptor complex member 2, which is involved in vesicle trafficking processes essential for cellular function. Identifying *GOSR2* mutations in patients with cardiac abnormalities can help understand the broader impact of vesicle trafficking pathways on heart structure and function [49]. This highlights the potential for cross-disciplinary approaches in managing patients with complex genetic profiles. *GOSR2* mutations are also linked to AF, coronary artery disease (CAD), myocardial infarction (MI), and HF, further emphasizing its systemic impact (Schmidt et al., 2023). Lahm et al. (2021) identified congenital heart disease risk loci through a genome-wide association study in European patients, which included significant associations with *GOSR2* mutations [50]. Their research provided insights into how disruptions in vesicle trafficking can affect cardiac development and function, contributing to a variety of cardiac conditions. The study emphasized that *GOSR2* mutations impair normal vesicle transport within cells, leading to defects in cardiac muscle cells that manifest as arrhythmias and structural heart diseases [50]. Additionally, *GOSR2*'s association with coronary artery disease (CAD) and myocardial infarction (MI) underscores its importance in vascular health. The gene's role in vesicle trafficking may influence the integrity of endothelial cells and smooth muscle cells in the vasculature. This can affect the development of atherosclerosis and the stability of atherosclerotic plaques, contributing to the risk of CAD and MI [50]. The identification of *GOSR2* mutations is clinically relevant for early diagnosis and intervention in patients with complex genetic profiles. Genetic screening for *GOSR2* variants in individuals presenting with unexplained cardiac and neurological symptoms can facilitate comprehensive management plans that address both sets of conditions. Understanding the systemic impact of *GOSR2* mutations can also inform the development of targeted therapies that aim to restore normal vesicle trafficking and cellular function.

***MYL4***

*MYL4* mutations are linked to various forms of congenital heart disease and cardiomyopathies [51]. *MYL4* encodes myosin light chain 4, which plays a crucial role in cardiac muscle contraction and atrial function. Genetic testing for *MYL4* can aid in diagnosing and managing atrial function disorders, specifically those related to atrial contraction and structure [51]. These findings align with previous reports associating *MYL4* mutations with AF and other atrial pathologies, emphasizing the need for targeted genetic screening [51,52]. Liu et al. (2022) investigated the relationship between serum miR-106 and *MYL4* levels and the prevalence, risk stratification, and prognosis of atrial fibrillation [52]. Their study demonstrated that elevated levels of *MYL4* are significantly associated with AF, suggesting that *MYL4* mutations contribute to atrial dysfunction by affecting the contractile properties of atrial myocytes [52]. The research highlighted that *MYL4* mutations can lead to impaired atrial contraction, which predisposes individuals to AF and other atrial arrhythmias. *MYL4* has also proved critical for the structural integrity and function of atrial myocytes. Mutations in *MYL4* disrupt the normal assembly and function of myosin filaments, leading to defective atrial contraction and atrial dilation. This structural impairment can result in a range of atrial pathologies, including atrial septal defects and atrial cardiomyopathies, which contribute to the development of AF and other arrhythmias [53]. The clinical implications of *MYL4* mutations extend to the management of atrial function disorders. Identifying *MYL4* mutations through genetic testing allows for early intervention and personalized treatment plans. These plans may include regular cardiac monitoring, pharmacological treatments to manage AF, and lifestyle modifications to reduce the risk of arrhythmias [52].

***ANKRD1***

The *ANKRD1* gene regulates the expression of cardiac-specific genes involved in response to mechanical stress. *ANKRD1* encodes the ankyrin repeat domain-containing protein 1, which plays a crucial role in cardiac muscle function and the stress response pathway. Mutations in *ANKRD1* have been linked to dilated cardiomyopathy (DCM) and HCM [54]. Identifying *ANKRD1* mutations may help in the early diagnosis and management of these cardiomyopathies, providing insights into potential therapeutic targets to modulate the cardiac stress response pathway [54]. Piroddi et al. (2020) investigated the effects of myocardial overexpression of *ANKRD1* and its impact on cardiac structure and function [55]. The study demonstrated that overexpression of *ANKRD1* in the myocardium leads to sinus venosus defects and progressive diastolic dysfunction. This suggests that *ANKRD1* plays a critical role in maintaining normal cardiac structure and function, and that its dysregulation can result in significant cardiac abnormalities [55]. The research highlighted that *ANKRD1* mutations can disrupt normal cardiac gene expression and protein interactions, leading to impaired cardiac function and the development of cardiomyopathies. Maron et al. (2012) explored *ANKRD1*’s involvement in the regulation of the cardiac stress response pathway [56]. It was found that mutations in *ANKRD1* can alter the expression of stress-responsive genes and proteins, leading to maladaptive cardiac remodelling and impaired contractility. This is particularly relevant in the context of DCM and HCM, where mechanical stress and hypertrophic signalling pathways are disrupted [56]. The ability of *ANKRD1* to modulate the cardiac stress response corroborates its potential as a therapeutic target for managing cardiomyopathies. The clinical implications of *ANKRD1* mutations extend to the diagnosis and management of cardiomyopathies. Genetic testing for *ANKRD1* variants in patients with a family history of DCM or HCM can facilitate early intervention and personalized treatment plans. These plans may include regular cardiac monitoring, pharmacological treatments to manage stress-induced cardiac dysfunction, and lifestyle modifications to reduce cardiac stress [54,55].

**Supplementary Figure 1.** Manhattan style plots listing genes prioritised for each left and right ventricular trait in the studies reviewed. Top row denotes the chromosome number and bottom row lists the gene name, whereas the vertical column lists the number of times the gene is prioritised.

**Supplementary Figure 2.** Manhattan style plots listing genes prioritised for each aorta, left and right atrium in the studies reviewed. Top row denotes the chromosome number and bottom row lists the gene name, whereas the vertical column lists the number of times the gene is prioritised.

**REFERENCES**

1. Gomes, B., Singh, A., O’sullivan, J. W., Schnurr, T. M., Goddard, P. C., Loong, S., Amar, D., Hughes, J. W., Kostur, M., Haddad, F., Salerno, M., Foo, R., Montgomery, S. B., Parikh, V. N., Meder, B., & Ashley, E. A. (2024). Genetic architecture of cardiac dynamic flow volumes. *Nature Genetics |*, *56*, 245–257. https://doi.org/10.1038/s41588-023-01587-5
2. Tharp, C. A., Haywood, M. E., Sbaizero, O., Taylor, M. R. G., & Mestroni, L. (2019). The Giant Protein Titin’s Role in Cardiomyopathy: Genetic, Transcriptional, and Post-translational Modifications of *TTN* and Their Contribution to Cardiac Disease. *Frontiers in Physiology*, *10*. https://doi.org/10.3389/FPHYS.2019.01436
3. Herman, D. S., Lam, L., Taylor, M. R. G., Wang, L., Christodoulou, D., Conner, L., Depalma, S. R., Mcdonough, B., Sparks, E., Lin Teodorescu, D., Cirino, A. L., Banner, N. R., Pennell, D. J., Graw, S., Merlo, M., Lenarda, A. Di, Sinagra, G., Martijn Bos, J., Ackerman, M. J., … Seidman, C. E. (2012). Truncations of Titin Causing Dilated Cardiomyopathy A BS T R AC T. *N Engl j Med*, *366*. [www.uniprot.org](http://www.uniprot.org)
4. Roberts, A. M., Ware, J. S., Herman, D. S., Schafer, S., Baksi, J., Bick, A. G., Buchan, R. J., Walsh, R., John, S., Wilkinson, S., Mazzarotto, F., Felkin, L. E., Gong, S., Macarthur, J. A. L., Cunningham, F., Flannick, J., Gabriel, S. B., Altshuler, D. M., MacDonald, P. S., … Cook, S. A. (2015). Integrated allelic, transcriptional, and phenomic dissection of the cardiac effects of titin truncations in health and disease. *Science Translational Medicine*, *7*(270), 270ra6.
5. Norton, N., Li, D., Rieder, M. J., Siegfried, J. D., Rampersaud, E., Züchner, S., Mangos, S., Gonzalez-Quintana, J., Wang, L., McGee, S., Reiser, J., Martin, E., Nickerson, D. A., & Hershberger, R. E. (2011). Genome-wide Studies of Copy Number Variation and Exome Sequencing Identify Rare Variants in *BAG3* as a Cause of Dilated Cardiomyopathy. *American Journal of Human Genetics*, *88*(3), 273. <https://doi.org/10.1016/J.AJHG.2011.01.016>
6. Knezevic, T., Myers, V. D., Gordon, J., Tilley, D. G., Thomas, •, Sharp Iii, E., Wang, J., Kamel Khalili, •, Cheung, J. Y., & Feldman, A. M. (n.d.). *BAG3*: a new player in the heart failure paradigm. *Heart Failure Reviews*, *20*. https://doi.org/10.1007/s10741-015-9487-6
7. Ying, Z.-M., Lv, Q.-K., Yao, X.-Y., Dong, A.-Q., Yang, Y.-P., Cao, Y.-L., Wang, F., Gong, A.-P., & Liu, C.-F. (2022). *BAG3* promotes autophagy and suppresses NLRP3 inflammasome activation in Parkinson’s disease. *Annals of Translational Medicine*, *10*(22), 1218–1218. https://doi.org/10.21037/ATM-22-5159
8. Sun, S., Shen, Y., Wang, J., Li, J., Cao, J., & Zhang, J. (2021). *Identification and Validation of Autophagy-Related Genes in Chronic Obstructive Pulmonary Disease*. https://doi.org/10.2147/COPD.S288428
9. Tcheandjieu, C., Xiao, K., Tejeda, H., Lynch, J. A., Ruotsalainen, S., Bellomo, T., Palnati, M., Judy, R., Klarin, D., Kember, R. L., Verma, S., Abecasis, G., Baras, A., Cantor, M., Coppola, G., Deubler, A., Economides, A., Karalis, K., Lotta, L. A., … Priest, J. R. (n.d.). *High heritability of ascending aortic diameter and trans-ancestry prediction of thoracic aortic disease*. <https://doi.org/10.1038/s41588-022-01070-7>
10. Milewicz, D. M., Kwartler, C. S., Papke, C. L., Regalado, E. S., Cao, J., & Reid, A. J. (2010). Genetic variants promoting smooth muscle cell proliferation can result in diffuse and diverse vascular diseases: Evidence for a hyperplastic vasculomyopathy. *Genetics in Medicine*, *12*(4), 196–203. <https://doi.org/10.1097/GIM.0B013E3181CDD687>
11. Francis, C. M., Futschik, M. E., & Huang, J. (n.d.-a). Enrico Petretto 14. *Mohammed-Aslam Imtiaz*, *10*, 34. https://doi.org/10.1038/s41467-022-32219-x
12. Li, N., Subrahmanyan, L., Smith, E., Yu, X., Zaidi, S., Choi, M., Mane, S., Nelson-Williams, C., Bahjati, M., Kazemi, M., Hashemi, M., Fathzadeh, M., Narayanan, A., Tian, L., Montazeri, F., Mani, M., Begleiter, M. L., Coon, B. G., Lynch, H. T., … Mani, A. (2016). Mutations in the Histone Modifier *PRDM6* Are Associated with Isolated Nonsyndromic Patent Ductus Arteriosus. *American Journal of Human Genetics*, *98*(6), 1082–1091. https://doi.org/10.1016/J.AJHG.2016.03.022
13. Yu, M., Harper, A., Yu, M., Harper, A. R., Aguirre, M., Pittman, M., Tcheandjieu, C., Amgalan, D., Grace, C., Goel, A., Farrall, M., Xiao, K., Engreitz, J., Pollard, K. S., Watkins, H., & Priest, J. R. (2023c). Circulation: Genomic and Precision Medicine 207 Genetic Determinants of the Interventricular Septum Are Linked to Ventricular Septal Defects and Hypertrophic Cardiomyopathy. *Circ Genom Precis Med*, *16*, 3708. https://doi.org/10.1161/CIRCGEN.122.003708
14. Aung, N., Vargas, J. D., Yang, C., Cabrera, C. P., Warren, H. R., Fung, K., Tzanis, E., Barnes, M. R., Rotter, J. I., Taylor, K. D., Manichaikul, A. W., Lima, J. A. C., Bluemke, D. A., Piechnik, S. K., Neubauer, S., Munroe, P. B., & Petersen, S. E. (2019). Genome-Wide Analysis of Left Ventricular Image-Derived Phenotypes Identifies Fourteen Loci Associated With Cardiac Morphogenesis and Heart Failure Development. *Circulation*, *140*(16), 1318–1330. <https://doi.org/10.1161/CIRCULATIONAHA.119.041161>
15. Ochoa, J. P., Sabater-Molina, M., García-Pinilla, J. M., Mogensen, J., Restrepo-Córdoba, A., Palomino-Doza, J., Villacorta, E., Martinez-Moreno, M., Ramos-Maqueda, J., Zorio, E., Peña-Peña, M. L., García-Granja, P. E., Rodríguez-Palomares, J. F., Cárdenas-Reyes, I. J., de la Torre-Carpente, M. M., Bautista-Pavés, A., Akhtar, M. M., Cicerchia, M. N., Bilbao-Quesada, R., … Monserrat, L. (2018). Formin Homology 2 Domain Containing 3 (*FHOD3*) Is a Genetic Basis for Hypertrophic Cardiomyopathy. *Journal of the American College of Cardiology*, *72*(20), 2457–2467. https://doi.org/10.1016/J.JACC.2018.10.001
16. Labat‐de‐hoz, L., & Alonso, M. A. (2021). Formins in Human Disease. *Cells*, *10*(10). https://doi.org/10.3390/CELLS10102554
17. Yu, M., Harper, A., Yu, M., Harper, A. R., Aguirre, M., Pittman, M., Tcheandjieu, C., Amgalan, D., Grace, C., Goel, A., Farrall, M., Xiao, K., Engreitz, J., Pollard, K. S., Watkins, H., & Priest, J. R. (2023a). Circulation: Genomic and Precision Medicine 207 Genetic Determinants of the Interventricular Septum Are Linked to Ventricular Septal Defects and Hypertrophic Cardiomyopathy. *Circ Genom Precis Med*, *16*, 3708. https://doi.org/10.1161/CIRCGEN.122.003708
18. Ye, J. Z., Delmar, M., Lundby, A., & Olesen, M. S. (2019). Reevaluation of genetic variants previously associated with arrhythmogenic right ventricular cardiomyopathy integrating population-based cohorts and proteomics data. *Clinical Genetics*, *96*(6), 506–514. https://doi.org/10.1111/CGE.13621
19. Grogan, A., & Kontrogianni-Konstantopoulos, A. (2019). Unraveling obscurins in heart disease. *Pflugers Archiv European Journal of Physiology*, *471*(5), 735–743. https://doi.org/10.1007/S00424-018-2191-3/METRICS
20. Marston, S., Li, A., dos Remedios, C. G., & Marston smarston, S. (2017). Obscurin variants and inherited cardiomyopathies. *Biophys Rev*, *9*, 239–243. https://doi.org/10.1007/s12551-017-0264-8
21. Levin, M. G., Tsao, N. L., Singhal, P., Liu, C., Vy, H. M. T., Paranjpe, I., Backman, J. D., Bellomo, T. R., Bone, W. P., Biddinger, K. J., Hui, Q., Dikilitas, O., Satterfield, B. A., Yang, Y., Morley, M. P., Bradford, Y., Burke, M., Reza, N., Charest, B., … Damrauer, S. M. (2022). Genome-wide association and multi-trait analyses characterize the common genetic architecture of heart failure. *Nature Communications*, *13*(1). <https://doi.org/10.1038/S41467-022-34216-6>
22. Yldau Van Der Ende, M., Said, A., Van Veldhuisen, D. J., Verweij, N., & Van Der Harst, P. (n.d.). *Genome-wide studies of heart failure and endophenotypes: lessons learned and future directions*. <https://doi.org/10.1093/cvr/cvy083>
23. Dávalos-Rodríguez, N. O., Rincón-Sánchez, A. R., Madrigal Ruiz, P. M., Flores-Alvarado, L. J., López-Toledo, S., Villafán-Bernal, J. R., Castro-Juárez, C. J., Guzmán-López, R., Siliceo-Murrieta, J. I., & Ramirez-Garcia, S. A. (2022). VNTR (CAG)n polymorphism of the *ATXN2* gene and metabolic parameters of cardiovascular risk associated with the degree of obesity in the Amerindian population of Oaxaca. *Endocrinología, Diabetes y Nutrición (English Ed.)*, *69*(1), 15–24. https://doi.org/10.1016/J.ENDIEN.2021.04.001
24. Li, P. P., Sun, X., Xia, G., Arbez, N., Paul, S., Zhu, S., Peng, H. B., Ross, C. A., Koeppen, A. H., Margolis, R. L., Pulst, S. M., Ashizawa, T., & Rudnicki, D. D. (2016). *ATXN2*-AS, a Gene Antisense to *ATXN2*, Is Associated with Spinocerebellar Ataxia Type 2 and Amyotrophic Lateral Sclerosis. *Annals of Neurology*, *80*(4), 600. https://doi.org/10.1002/ANA.24761
25. Meyer, H. V. (2020). Genetic and functional insights into the fractal structure of the heart. *Nature*, *584*. https://doi.org/10.1038/s41586-020-2635-8
26. Delisle, B. P., Yu, Y., Puvvula, P., Hall, A. R., Huff, C., & Moon, A. M. (2019). *TBX3*-Mediated Regulation of Cardiac Conduction System Development and Function: Potential Contributions of Alternative RNA Processing. *Pediatric Cardiology*, *40*(7), 1388–1400. https://doi.org/10.1007/S00246-019-02166-4/METRICS
27. Bakker, M. L., Boukens, B. J., Mommersteeg, M. T. M., Brons, J. F., Wakker, V., Moorman, A. F. M., & Christoffels, V. M. (2008). Transcription Factor *TBX3* Is Required for the Specification of the Atrioventricular Conduction System. *Circulation Research*, *102*(11), 1340–1349. https://doi.org/10.1161/CIRCRESAHA.107.169565
28. Stennard, F. A., & Harvey, R. P. (2005). T-box transcription factors and their roles in regulatory hierarchies in the developing heart. *Development*, *132*(22), 4897–4910. https://doi.org/10.1242/DEV.02099
29. Smemo, S., Campos, L. C., Moskowitz, I. P., Krieger, J. E., Pereira, A. C., & Nobrega, M. A. (2012). Regulatory variation in a *TBX5* enhancer leads to isolated congenital heart disease. *Human Molecular Genetics*, *21*(14), 3255–3263. <https://doi.org/10.1093/HMG/DDS165>
30. Steimle, J. D., & Moskowitz, I. P. (2017). *TBX5*: A Key Regulator of Heart Development. *Current Topics in Developmental Biology*, *122*, 195–221. https://doi.org/10.1016/BS.CTDB.2016.08.008
31. Van Ouwerkerk, A. F., Bosada, F. M., Van Duijvenboden, K., Houweling, A. C., Scholman, K. T., Wakker, V., Allaart, C. P., Uhm, J. S., Mathijssen, I. B., Baartscheer, T., Postma, A. V., Barnett, P., Verkerk, A. O., Boukens, B. J., & Christoffels, V. M. (2022). Patient-Specific *TBX5*-G125R Variant Induces Profound Transcriptional Deregulation and Atrial Dysfunction. *Circulation*, *145*(8), 606. https://doi.org/10.1161/CIRCULATIONAHA.121.054347
32. Aung, N., Lopes, L. R., Van Duijvenboden, S., Harper, A. R., Goel, A., Grace, C., Ho, C. Y., Weintraub, W. S., Kramer, C. M., Neubauer, S., Watkins, H. C., Petersen, S. E., & Munroe, P. B. (2023). Genome-Wide Analysis of Left Ventricular Maximum Wall Thickness in the UK Biobank Cohort Reveals a Shared Genetic Background With Hypertrophic Cardiomyopathy. *Circulation. Genomic and Precision Medicine*, *16*(1), E003716. https://doi.org/10.1161/CIRCGEN.122.003716
33. Cao, C., Li, L., Zhang, Q., Li, H., Wang, Z., Wang, A., & Liu, J. (2023). Nkx2.5: a crucial regulator of cardiac development, regeneration and diseases. *Frontiers in Cardiovascular Medicine*, *10*. https://doi.org/10.3389/FCVM.2023.1270951
34. Lemmens, R., Hermans, S., Nuyens, D., & Thijs, V. (2011). Access to. *Research Stroke Research and Treatment*, *2011*. https://doi.org/10.4061/2011/208694
35. *A comprehensive 1000 Genomes-based genome-wide association meta-analysis of coronary artery disease*. (2015). <https://doi.org/10.1038/ng.3396>
36. Zhang, J., Guo, Y., Zhao, X., Pang, J., Pan, C., Wang, J., Wei, S., Yu, X., Zhang, C., Chen, Y., Yin, H., & Xu, F. (2023). The role of aldehyde dehydrogenase 2 in cardiovascular disease. *Nature Reviews Cardiology |*, *20*, 495–509. https://doi.org/10.1038/s41569-023-00839-5
37. Zhang, Y., & Ren, J. (2011). *ALDH2* in alcoholic heart diseases: Molecular mechanism and clinical implications. *Pharmacology & Therapeutics*, *132*(1), 86–95. https://doi.org/10.1016/J.PHARMTHERA.2011.05.008
38. Roselli, C., Chaffin, M. D., Weng, L.-C., Aeschbacher, S., Ahlberg, G., Albert, C. M., Almgren, P., Alonso, A., Anderson, C. D., Aragam, K. G., Arking, D. E., Barnard, J., Bartz, T. M., Benjamin, E. J., Bihlmeyer, N. A., Bis, J. C., Bloom, H. L., Boerwinkle, E., Bottinger, E. B., … Ellinor, P. T. (n.d.). *Multi-ethnic genome-wide association study for atrial fibrillation*. https://doi.org/10.1038/s41588-018-0133-9
39. McBride, D., Deshmukh, A., Shore, S., Elafros, M. A., & Liang, J. J. (2022). Cardiac Involvement and Arrhythmias Associated with Myotonic Dystrophy. *Reviews in Cardiovascular Medicine*, *23*(4). https://doi.org/10.31083/J.RCM2304126
40. Ueda, H., Ohno, S., & Kobayashi, T. (2000). Myotonic Dystrophy and Myotonic Dystrophy Protein Kinase. *Progress in Histochemistry and Cytochemistry*, *35*(3), 187–251. https://doi.org/10.1016/S0079-6336(00)80002-9
41. Magnani, J. W., Yin, X., Mcmanus, D. D., Chuang, M. L., Cheng, S., Lubitz, S. A., Arora, G., Manning, W. J., Ellinor, P. T., & Benjamin, E. J. (n.d.). *Genetic Loci Associated With Atrial Fibrillation: Relation to Left Atrial Structure in the Framingham Heart Study*. https://doi.org/10.1161/JAHA.113.000616
42. Lauriol, J., & Kontaridis, M. I. (2011). *PTPN11*-associated mutations in the heart: has LEOPARD changed Its RASpots? *Trends in Cardiovascular Medicine*, *21*(4), 97–104. https://doi.org/10.1016/J.TCM.2012.03.006
43. Tartaglia, M., Gelb, B. D., & Zenker, M. (2011). Noonan syndrome and clinically related disorders. *Best Practice & Research Clinical Endocrinology & Metabolism*, *25*(1), 161–179. https://doi.org/10.1016/J.BEEM.2010.09.002
44. Pandit, B., Sarkozy, A., Pennacchio, L. A., Carta, C., Oishi, K., Martinelli, S., Pogna, E. A., Schackwitz, W., Ustaszewska, A., Landstrom, A., Bos, J. M., Ommen, S. R., Esposito, G., Lepri, F., Faul, C., Mundel, P., López Siguero, J. P., Tenconi, R., Selicorni, A., … Gelb, B. D. (2007). Gain-of-function RAF1 mutations cause Noonan and LEOPARD syndromes with hypertrophic cardiomyopathy. *Nature Genetics 2007 39:8*, *39*(8), 1007–1012. https://doi.org/10.1038/ng2073
45. Fox, E. R., Musani, S. K., Barbalic, M., Lin, H., Yu, B., Ogunyankin, K. O., Smith, N. L., Kutlar, A., Glazer, N. L., Post, W. S., Paltoo, D. N., Dries, D. L., Farlow, D. N., Duarte, C. W., Kardia, S. L., Meyers, K. J., Sun, Y. V., Arnett, D. K., Patki, A. A., … Vasan, R. S. (2013). Genome-Wide Association Study of Cardiac Structure and Systolic Function in African Americans: The Candidate Gene Association Resource (CARe) Study. *Circulation. Cardiovascular Genetics*, *6*(1), 37. https://doi.org/10.1161/CIRCGENETICS.111.962365
46. Merner, N. D., Hodgkinson, K. A., Haywood, A. F. M., Connors, S., French, V. M., Drenckhahn, J. D., Kupprion, C., Ramadanova, K., Thierfelder, L., McKenna, W., Gallagher, B., Morris-Larkin, L., Bassett, A. S., Parfrey, P. S., & Young, T. L. (2008). Arrhythmogenic Right Ventricular Cardiomyopathy Type 5 Is a Fully Penetrant, Lethal Arrhythmic Disorder Caused by a Missense Mutation in the *TMEM43* Gene. *American Journal of Human Genetics*, *82*(4), 809. https://doi.org/10.1016/J.AJHG.2008.01.010
47. Zink, M., Seewald, A., Rohrbach, M., Brodehl, A., Liedtke, D., Williams, T., Childs, S. J., & Gerull, B. (2022). Altered Expression of *TMEM43* Causes Abnormal Cardiac Structure and Function in Zebrafish. *International Journal of Molecular Sciences*, *23*(17). https://doi.org/10.3390/IJMS23179530
48. Lindy, A. S., Mary, |, Stosser, B., Butler, E., Downtain-Pickersgill, C., Shanmugham, A., Retterer, K., Brandt, T., Richard, G., Dianalee, |, & Mcknight, A. (2018). *Diagnostic outcomes for genetic testing of 70 genes in 8565 patients with epilepsy and neurodevelopmental disorders*. https://doi.org/10.1111/epi.14074
49. Schmidt, A. F., Bourfiss, M., Alasiri, A., Puyol-Anton, E., Chopade, S., van Vugt, M., van der Laan, S. W., Gross, C., Clarkson, C., Henry, A., Lumbers, T. R., van der Harst, P., Franceschini, N., Bis, J. C., Velthuis, B. K., te Riele, A. S. J. M., Hingorani, A. D., Ruijsink, B., Asselbergs, F. W., … Finan, C. (2023). Druggable proteins influencing cardiac structure and function: Implications for heart failure therapies and cancer cardiotoxicity. *Science Advances*, *9*(17). https://doi.org/10.1126/SCIADV.ADD4984
50. Lahm, H., Jia, M., Dreßen, M., Wirth, F., Puluca, N., Gilsbach, R., Keavney, B. D., Cleuziou, J., Beck, N., Bondareva, O., Dzilic, E., Burri, M., König, K. C., Ziegelmüller, J. A., Abou-Ajram, C., Neb, I., Zhang, Z., Doppler, S. A., Mastantuono, E., … Krane, M. (2021). Congenital heart disease risk loci identified by genome-wide association study in European patients. *The Journal of Clinical Investigation*, *131*(2). https://doi.org/10.1172/JCI141837
51. Thanaj, M., Mielke, J., McGurk, K. A., Bai, W., Savioli, N., Marvao, A., Meyer, H. V, Zeng, L., Sohler, F., Thomas Lumbers, R., Wilkins, M. R., Ware, J. S., Bender, C., Rueckert, D., MacNamara, A., Freitag, D. F., & ORegan, D. P. (n.d.). *Genetic and environmental determinants of diastolic heart function*. https://doi.org/10.1038/s44161-022-00048-2
52. Liu, Y., Zhou, H., Tao, Y., Xu, Z., & Lai, H. (2022). Relationship between Serum miR-106 and *MYL4* Levels and the Prevalence, Risk Stratification, and Prognosis of Atrial Fibrillation. *Journal of Immunology Research*, *2022*. https://doi.org/10.1155/2022/1069866
53. Odak, M., Douedi, S., Mararenko, A., Alshami, A., Elkherpitawy, I., Douedi, H., Zacks, E., & Sealove, B. (2022). Arrhythmogenic Right Ventricular Cardiomyopathy: The Role of Genetics in Diagnosis, Management, and Screening. *Cardiology Research*, *13*(4), 177–184. https://doi.org/10.14740/CR1373
54. Pirruccello, J. P., Rämö, J. T., Choi, S. H., Chaffin, M. D., Kany, S., Nekoui, M., Chou, E. L., Jurgens, S. J., Friedman, S. F., Juric, D., Stone, J. R., Batra, P., Ng, K., Philippakis, A. A., Lindsay, M. E., & Ellinor, P. T. (2023). The Genetic Determinants of Aortic Distention. *Journal of the American College of Cardiology*, *81*(14), 1320–1335. https://doi.org/10.1016/J.JACC.2023.01.044
55. Piroddi, N., Pesce, P., Scellini, B., Manzini, S., Ganzetti, G. S., Badi, I., Menegollo, M., Cora, V., Tiso, S., Cinquetti, R., Monti, L., Chiesa, G., Bleyl, S. B., Busnelli, M., Dellera, F., Bruno, D., Caicci, F., Grimaldi, A., Taramelli, R., … Campione, M. (2020). Myocardial overexpression of *ANKRD1* causes sinus venosus defects and progressive diastolic dysfunction. *CARDIOVASCULAR RESEARCH*, *116*(8), 1458–1472. https://doi.org/10.1093/CVR/CVZ291
56. Maron, B. J., Maron, M. S., & Semsarian, C. (2012). Genetics of Hypertrophic Cardiomyopathy After 20 Years: Clinical Perspectives. *Journal of the American College of Cardiology*, *60*(8), 705–715. https://doi.org/10.1016/J.JACC.2012.02.068
